# Gold Nanoparticles: Tunable Characteristics and Potential for Nasal Drug Delivery

**DOI:** 10.3390/pharmaceutics16050669

**Published:** 2024-05-16

**Authors:** Aida Maaz, Ian S. Blagbrough, Paul A. De Bank

**Affiliations:** 1 Department of Life Sciences, University of Bath, Bath BA2 7AY, UK; 2 Centre for Therapeutic Innovation, University of Bath, Bath BA2 7AY, UK; 3 Centre for Bioengineering & Biomedical Technologies, University of Bath, Bath BA2 7AY, UK

**Keywords:** gold nanoparticles, intranasal, nose-to-brain, pMDI, RPMI 2650

## Abstract

A general procedure to prepare gold nanourchins (GNUs) via a seed-mediated method was followed using dopamine hydrochloride as a reducing agent and silver nitrate salt (AgNO_3_) as a shape-directing agent. The novelty of this study comes from the successful incorporation of the prepared gold urchins as an aqueous suspension in a nasal pressurized metered dose inhaler (pMDI) formulation and the investigation of their potential for olfactory targeting for direct nose-to-brain drug delivery (NTBDD). The developed pMDI formulation was composed of 0.025% *w*/*w* GNUs, 2% *w*/*w* Milli-Q water, and 2% *w*/*w* EtOH, with the balance of the formulation being HFA134a propellant. Particle integrity and aerosolization performance were examined using an aerosol exposure system, whereas the nasal deposition profile was tested in a sectioned anatomical replica of human nasal airways. The compatibility of the gold dispersion with the nasal epithelial cell line RPMI 2650 was also investigated in this study. Colloidal gold was found to be stable following six-month storage at 4 °C and during the lyophilization process utilizing a pectin matrix for complete re-dispersibility in water. The GNUs were intact and discrete following atomization via a pMDI, and 13% of the delivered particles were detected beyond the nasal valve, the narrowest region in the nasal cavity, out of which 5.6% was recovered from the olfactory region. Moreover, the formulation was found to be compatible with the human nasal epithelium cell line RPMI 2650 and excellent cell viability was observed. The formulated GNU-HFA-based pMDI is a promising approach for intranasal drug delivery, including deposition in the olfactory region, which could be employed for NTBDD applications.

## 1. Introduction

Gold nanoparticles (GNPs) have become attractive tools in sensing [1], imaging [2,3], tissue engineering [4], gene and drug delivery [5], and as theranostic carriers [6] due to their tunable properties, including morphology, optical characteristics, high stability, and easy conjugation or functionalization with small- as well as macro-molecules. GNPs have the potential for targeted delivery to cells or tissues of interest, achieving high levels of uptake and overcoming multi-drug resistance (MDR) in tumour cells [7]. Despite the incomplete understanding of the long-term biological fate of GNPs and a perception that gold’s inertness may limit their biodegradation, a recent study demonstrated the intracellular metabolism of GNPs via a two-step degradation and recrystallization mechanism [8].

A significant feature of GNPs, which has resulted in an expanding range of biomedical applications such as diagnostics and therapeutics, is their strong localized surface plasmon resonance (LSPR). The optical characteristics of gold particles are a consequence of oscillations in surface electrons when exposed to electromagnetic waves at specific frequencies. LSPR is dependent on the physical properties of the GNPs, including size, shape, crystal structure, surface functionalization and the dielectric environment [9]. As such, while the corresponding LSPR for a specific GNP size and shape could be clearly observed in the visible region of the spectrum (500–600 nm), highly branched anisotropic GNPs exhibit a longitudinal LSPR maximum that shifts into the near-infrared (NIR) region. A NIR laser could take advantage of this bio-transparent window between 780 and 1000 nm to selectively photothermally kill tumour cells which have internalized GNPs with minimal damage to healthy tissues [10]. A further attractive feature of GNPs is that they can be readily functionalized with various molecules, including peptides, proteins, and antibodies, to target specific cells or tissues of interest [11,12]. Combined with their optical responsiveness, GNPs are an attractive prospect for the development of targeting theranostic systems. 

GNPs have also been investigated as nanocarriers with suitability for brain targeting via nasal delivery [13,14,15,16]. The intranasal (IN) delivery route is emerging as a therapeutic avenue for various central nervous system (CNS) disorders, with therapeutic molecules able to directly access the brain via olfactory and trigeminal pathways in the nasal cavity, circumventing the blood–brain barrier, which limits drug access to the CNS via systemic delivery [17]. In addition to the potential of nose-to-brain drug delivery (NTBDD), the IN route offers several advantages, including a large surface area, rapid onset of action, ease of administration and non-invasiveness. IN delivery is increasingly considered when developing new therapies and drug delivery systems. For example, Wang et al. developed temozolomide-conjugated GNPs, which were functionalized with an anti-EphA3 antibody to specifically target tumour cells and overcome glioblastoma MDR following IN administration [16]. Betzer et al. demonstrated that glucose-functionalized GNPs with a diameter of 5 nm improved the brain delivery and tracking of exosomes delivered via the IN route for diagnostic purposes and, potentially, as therapies for various CNS disorders [3]. Similarly, GNPs have been used to track resveratrol-loaded transferosomes for the potential treatment of Alzheimer’s disease following IN administration. Histology and computed tomography demonstrated the accumulation of GNPs in the brain tissue of treated animals [18]. 

For NTBDD, the deposition and retention of nanoparticles in the nasal cavity and their subsequent translocation towards the brain are highly dependent on the particle size and inhalation exposure time [19]. Gallardo-Toledo et al. demonstrated that different GNP morphologies (i.e., size and shape) behaved differently when evaluated for nose-to-CNS delivery. Gold nanospheres (47 nm) achieved higher concentrations in brain tissue than gold nanoprisms (78 nm). Moreover, the levels of gold nanospheres were 55-fold higher following IN delivery in comparison to intravenous injection [14]. Despite its significant potential, efficient brain targeting via IN administration is challenging. The human nose has extremely complex cross-sectional geometries, and the olfactory region, the area of the nasal cavity targeted for NTBDD, covers a relatively small surface area (<10%) at the top of the cavity [20]. Hence, the nasal application of formulations requires deep penetration to the internal nasal structures if therapeutic levels of drug are to be achieved. An alternative approach to traditional nasal formulations should reduce the quantity of formulation that escapes the nasal passage and is swallowed, reduce off-nose deposition, and enhance posterior delivery, bypassing the nasal valve. We hypothesize that pressurized metered-dose inhalers (pMDIs), which have been traditionally used for the treatment of asthma and other pulmonary disorders, could be suitable devices to achieve this aim by delivering aerosolized GNPs deep into the nasal cavity. 

Hence, the aim of this study was to explore the nasal delivery of intact and respirable GNPs via a pMDI device and assess the potential of the developed system to offer deep penetration of the formulation into the nasal cavity. Specifically, we focussed on the generation of anisotropic gold nanourchins (GNUs), which can be produced via facile aqueous chemical synthesis. These nanoparticles have a relatively high aspect ratio, and LSPR shifted towards the NIR region to take advantage of the limited absorption of these wavelengths by biological tissues for future applications in NTBDD and diagnostics. To this end, the deposition pattern of GNUs delivered using a pMDI was examined in vitro using a 3D-printed replica of the human nasal cavity, and the compatibility of the GNU formulation with the RPMI 2650 nasal epithelial cell line was investigated.

## 2. Materials and Methods

### 2.1. Materials

Hydrogen tetrachloroaurate (III) trihydrate (HAuCl_4_·3H_2_O), dopamine hydrochloride (DA), hydroquinone (HQ), sodium citrate dihydrate (C_6_H_5_Na_3_O_7_·2H_2_O), poly(ethylene glycol) methyl ether thiol (mPEG thiol, average Mn 6 kDa), silver nitrate (AgNO_3_), apple pectin, porcine stomach mucin-type II, Tween^®^80, hydrochloric acid (HCl, 34–37%) and nitric acid (HNO_3_, 68%) for trace metal analysis were purchased from Sigma-Aldrich (Gillingham, Dorset, UK). TraceCERT^®^, the gold standard for ICP, 1000 mg/L Au in hydrochloric acid, was purchased from Merck. All glassware and magnetic stirrer bars were rigorously cleaned with aqua regia (HCl:HNO_3_ 3:1 *v*/*v*) and then thoroughly rinsed with Milli-Q water (18.2 MΩ·cm resistivity at 22 °C) before use. For the cell work, the RPMI 2650 cell line was purchased from ECACC (European Collection of Authenticated Cell Cultures, ECACC 88031602, Porton Down, UK). Eagle’s minimal essential medium (EMEM) with L-glutamine, heat-inactivated foetal bovine serum (FBS), non-essential amino acids solution (NEAA), penicillin/streptomycin antibiotic solution and Hank’s balanced salt solution (HBSS) were purchased from Sigma Aldrich (Gillingham, Dorset, UK).

### 2.2. Preparation of Gold Urchin-like Nanocrystals (GNUs)

To produce quality GNUs with high monodispersity, the reduction of AuCl_4_^−^ was performed in the presence of pre-synthesized spherical gold seeds, which were prepared via the citrate reduction method reported in [21] and further developed in [22]. The Au seeds acted as catalyst centres that enabled the reduction and selective accumulation of gold atoms on the seeds’ surfaces by the reducing agent, thus preventing secondary nucleation [23]. In a typical reaction, 100 mL of 0.3 mM (0.04%) HAuCl_4_·3H_2_O aq. was heated under reflux for 20 min and 300 µL of 0.388 M (11.4%) sodium citrate dihydrate then added under continuous stirring at 800 rpm. A red/orange-coloured solution was observed within 15 min of heating, indicating seed formation. The citrate-capped gold seeds were purified from excess reagents via three cycles of pelleting by centrifugation at 20,000 rpm for 2 h (Beckman J2, fixed angle rotor JA-20.1, Beckman Coulter, Brea, CA, USA) and washing with Milli-Q water. 

The preparation of GNUs followed a method described by Ong et al. [11]. In a 10 mL vial of ultra-pure water, maintained at 30 ± 1 °C in an oil bath, 20 µL of 0.1 M gold salt HAuCl_4_·3H_2_O, 100 µL of 0.1 mM AgNO_3_, 60 µL of citrate capped seeds, 80 µL of 1.7 mM mPEG thiol and different volumes of either 15 mM HQ (100, 300, 500 and 1000 µL) or 15 mM DA (300, 600, 800 and 1000 µL) were subsequently added and the mixture was then thoroughly stirred at 800 rpm and allowed to react for 2 h. The resulting dark-blue-coloured solution was indicative of the successful nucleation and growth of gold particles, which were then purified from excess reagents via centrifugation at 800× *g* for 10 min and washing 3 times with Milli-Q water. For GNU synthesis, the effect of some reaction parameters, such as PEGylation and the Ag:Au ratio (1:0, 1:10, 1:100, 1:500 and 1:1000) on the growth of particles and their final morphology was investigated. Finally, for lyophilization, 1 mL of the optimized GNU formulation containing 14% *v*/*v* of 0.25% *w*/*v* pectin solution as a cryoprotectant [24] was snap-frozen using liquid nitrogen. The frozen suspension within a pectin matrix was then freeze-dried using a LyoQuest-55 freeze-dryer (Azbil Telstar Technologies, Barcelona, Spain) at −50 °C under vacuum (0.1 bar) for at least 16 h. The resulting dry matter was used to formulate a nanosuspension-based pMDI formulation.

### 2.3. Characterization of GNUs

Particle size distribution, polydispersity index (PDI) and zeta potential of gold colloid dispersions were measured via dynamic light scattering (DLS) using a Malvern Zetasizer Nano ZS90 (Malvern Instrument, Malvern, UK) equipped with a He-Ne laser, at a scattering angle of 173° at 25 °C. Measurements were conducted in three replicates per sample and recorded as the mean ± SD of three runs. The size distribution was further investigated through the use of nanoparticle tracking analysis (NTA) using a NanoSight NS500 (Malvern Instrument, Malvern, UK). At least five recordings per sample were performed, and the data were acquired using NanoSight NTA software v3.40 and reported as the mean ± SD. The synthesis of GNUs was confirmed via UV-Vis spectrophotometry (Jenway 6850, Cole-Parmer, St Neots, UK), observing the LSPR band for the optimized GNU formulation. Particle imaging before and after filling pMDI devices was performed using transmission electron microscopy (TEM) (JEOL JEM-2100 Plus, JEOL Ltd., Akishima, Japan) operating at an acceleration voltage of 200 kV. The samples were prepared by placing a drop of gold suspension on a carbon-coated copper grid and placing under vacuum for 16 h prior to imaging. Aerosol samples were sprayed directly onto the grid, which was placed on a receiver membrane in a sample collection apparatus. The grids were then removed and similarly placed under vacuum for 16 h prior to imaging. A mica substrate was also place on the receiver membrane to collect aerosol samples. After drying under vacuum for 16 h, mica substrates were coated with a 10 nm layer of chromium (Edwards sputter coater, S150B, Edwards Vacuum, Burgess Hill, UK) and subsequently imaged using field emission-scanning electron microscopy (FE-SEM) (JEOL JSM-6301F, JEOL Ltd., Akishima, Japan) at an acceleration voltage of 5 kV. SEM and energy-dispersive X-ray (EDX) analysis was also conducted to investigate the presence of elemental gold on the membrane. Quantitative analysis of elemental gold to screen the deposited dose of GNUs in the nasal cast was carried out using inductively coupled plasma-optical emission spectroscopy (ICP-OES, Agilent Technologies, Santa Clara, CA, USA) operating with ICP Expert II Software, v2.0.4. A calibration curve was generated from six aqueous gold calibration standards over the range of 0.05–2 ppm. 

### 2.4. Gold Colloidal Dispersions in HFA134a

The optimized GNU formulation was prepared within a pectin matrix and lyophilized as described in Section 2.2 prior to redispersion in the pMDI formulation. Throughout the work, pMDI devices were prepared by an expert team on-site at Nanopharm Ltd. (Newport, UK). The GNUs (0.025% *w*/*w* of the total pMDI formulation mass) were redispersed in Milli-Q water (2% *w*/*w*) within a 17 mL uncoated aluminium canister followed by sonication for 3 min (Ultrasonic bath USC, VWR, Lutterworth, UK). EtOH (2% *w*/*w*) was subsequently added and mixed thoroughly by vortexing for 30 s. The canisters were crimped with a 50 µL EPDM Aptar valve using a manual bench-top crimper (Laboratory Plant 2016, Pamasol Willi Mäder AG, Pfäffikon, Switzerland). The balance of HFA134a propellant was filled through the valve using HFA134a propellant filler, and the final product was vortexed for 90 s. The canisters were then kept inverted (i.e., valves down) for 72 h at 20 °C to allow valve expansion prior to aerosol performance testing.

### 2.5. Shot Weight Measurements

Shot weight is defined by the mass difference between two actuations of the pMDI. In a general procedure, pMDI canisters were manually shaken 3 to 5 times, and three actuations were primed to waste prior to the test. Atomization was performed with the same printed actuator used for the deposition studies. An accurate analytical balance (VMP0281, Sartorius, Göttingen, Germany) was utilized to record the device weight, and thus the actuated mass, before and after each experiment to confirm the delivered mass uniformity throughout the study.

### 2.6. GNU Integrity in pMDI Formulation

**Figure 1:**
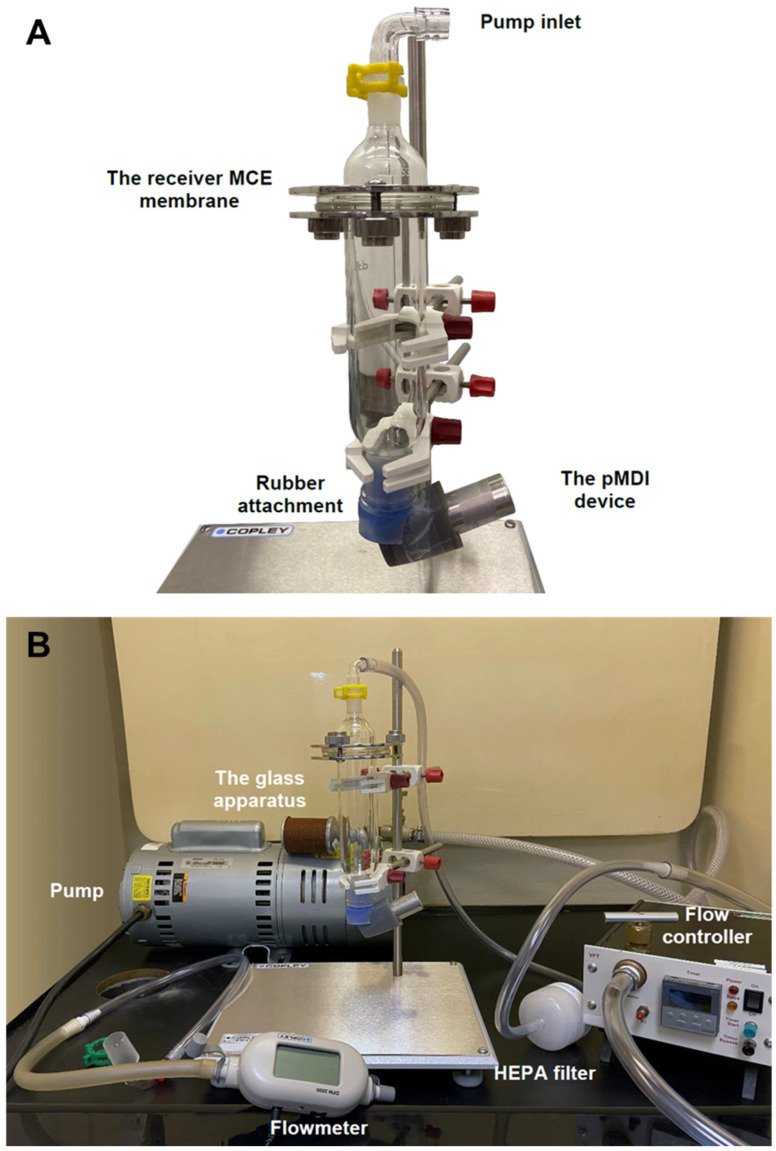
(**A**) The glass apparatus used to contain the aerosolized particles. (**B**) The aerosol exposure system components and accessories.

To test the integrity of GNUs in terms of size and shape within the formulation, particles were collected from the spray mist using an aerosol exposure system consisting of a glass apparatus (Sample Collection Apparatus for FP/Salmeterol Powders, Cat. No. 8640, Copley Scientific, Nottingham, UK), which is normally used for dose uniformity tests for inhaled powders. The apparatus was fixed upside down as its intended use was to simulate nasal delivery, as demonstrated in Figure 1A. A mixed cellulose ester (MCE) membrane (Millipore, Burlington, MA, USA; 1.2 µm pore size) was placed at the top of the glass tube to receive the fired shot. To replicate moderate human nasal breathing (9–20 L/min for adults) [25], a vacuum pump was connected to the top of the apparatus to generate an inspiratory flow, and a flow controller and a flow meter were used to manually adjust the airflow to a 15 ± 0.2 L/min (Figure 1B). A piece of mica and a carbon-coated grid were carefully placed on the membrane to collect particles for imaging via SEM and TEM, respectively. Prior to aerosolization, the canisters were shaken ~10 times, sonicated for 90 s and finally primed to waste three times. Thirty actuations were carried out per test.

### 2.7. Nasal Deposition of Aerosolized GNUs

**Figure 2:**
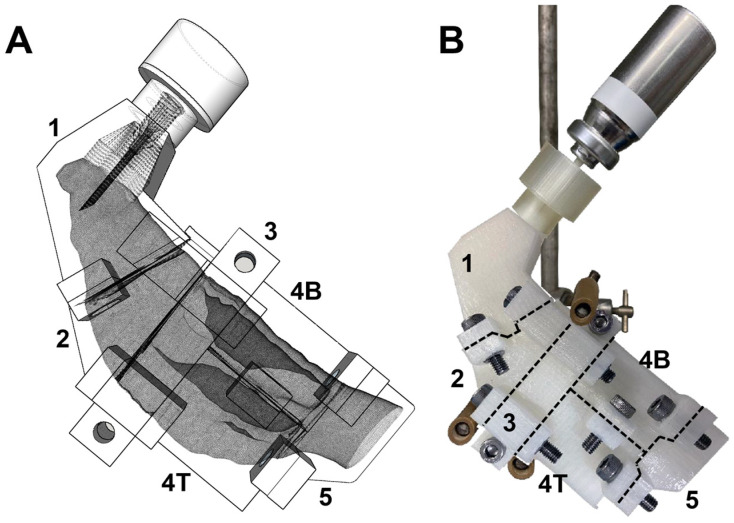
(**A**) The 3D design of the nasal cast with a closed outlet to mimic no airflow condition and demonstrate the position of the needle used to deliver the formulation, (**B**) the nylon 3D-printed cast positioned at 120° to the horizontal plane with the delivery device in place. Sections are numbered as follows: 1: the vestibule; 2: the nasal valve; 3: the front turbinates; 4T: the olfactory region; 4B: the rear turbinates; and 5: the nasopharynx.

The regional deposition of aerosolized GNUs was investigated using a seven-sectioned 3D nasal model based on the Carleton-Civic standardized human nasal geometry developed by Liu et al. [26] and sectioned using planar section cuts in a similar manner to the nasal casts reported by Hughes et al. [27] and Schroeter et al. [28]. The resulting olfactory region of the model contained a portion of the superior turbinate and meatus and had a total surface area of 24.3 cm^2^. The 3D model of the nasal replica was generated using SketchUp (Trimble Inc., Westminster, CO, USA; Figure 2A) and was printed using fused deposition modelling printing technology (Ultimaker 2+) using polyamide (nylon) as the printing material (Figure 2B). A bespoke, in-house 3D-printed nasal device made of polylactic acid (PLA) and attached to a needle (1.6 mm O.D. × 40 mm) was used for formulation delivery, acting as an adapter between the pMDI canister and the nasal replica. Using a brush, the inner surface of each section of the cast was coated with 4% *w*/*v* mucin-based mucus simulant. The mucus simulant was prepared by suspending lyophilized type III porcine gastric mucin in isotonic phosphate buffer (pH 6.5) containing 0.02% *w*/*v* sodium azide as a preservative. Undissolved residues were removed by centrifugation at 800× *g* for 1 h (Beckman J2, Beckman Coulter, Brea, CA, USA). The reconstituted mucus solutions were stored at −20 °C and thawed before use. All deposition experiments were carried out via manual actuation, and repeated shaking of canisters by hand (~10 times) between actuations was applied to avoid formulation creaming effects, thus maximizing dose uniformity. Following the assembly of the cast sections with nuts and bolts, silicon grease (Korasilon^®^ medium viscosity) was applied to coat the anterior and posterior surfaces of each section to ensure a tight seal. For the deposition experiments, the cast was clamped at 120° to the horizontal plane (Figure 2B). Thirty shake actuation cycles were performed with a 5 s flush time and no airflow was applied. To achieve this, the distal face of section 5 of the cast, which represents the back of the entrance to the nasopharynx, was sealed. Following each run, the cast was disassembled, and each section was rinsed thoroughly with up to 10 mL of an aqueous 0.01% *w*/*v* solution of Tween^®^80 using a pipette. The wash samples from each section were then dried using a rotary evaporator (Rotavapor R II with a V-700 vacuum pump, BŰCHI, Flawil, Switzerland) to prepare the samples for digestion.

ICP-OES was used to determine the quantity of elemental gold in each section of the cast. The GNUs and any organic residues from each dried sample were digested by adding 1 mL of aqua regia and heating the samples to 90 °C until complete evaporation. This was repeated until no particulates remained. The samples were finally prepared for analysis via further dilution with 1 M HNO_3_ to a volume of 5 mL with a final aqua regia concentration of 10% *v*/*v*. A blank sample was also prepared by adding 150 µL mucus simulant to a glass vial and following the same digestion procedure used for the gold samples. The absorbance of the samples was measured, and the gold concentrations in each section were determined by comparison with a calibration curve of gold solutions with known concentrations (50, 100, 500, 1000, 1500 and 2000 µg/L). The lower limit of detection for the ICP-OES was found to be 0.05 µg/L.

### 2.8. RPMI 2650 Cell Culture Maintenance

RPMI 2650 nasal epithelial cells were initially cultured according to the ECACC protocol in 75 cm^2^ flasks using a seeding density of 2–4 × 10^3^ cells/cm^2^ and culture medium consisting of EMEM supplemented with 10% FBS, 1% L-glutamine, 1% NEAA and 1% penicillin/streptomycin antibiotic mixture. The cells were incubated at 37 °C in a humidified 5% CO_2_ atmosphere and passaged when ~80% confluent (every 5 to 6 days).

### 2.9. In Vitro Cytotoxicity of GNUs

The compatibility of the GNU formulation with RPMI 2650 cells was assessed using the resazurin cell metabolism assay. RPMI 2650 cells were seeded in 96-well plates at 5 × 10^3^ cells/well and cultured for 96 h. An aqueous 1% *w*/*v* GNU suspension was generated from the pectin-based lyophilizate (Section 2.2) and diluted with cell culture medium to concentrations between 0.01% and 0.08% *w*/*v*, which were maintained for 24 h at 25 °C. The cells were then incubated with 100 µL of the pre-equilibrated NP suspensions for 4 or 24 h at 37 °C, 5% CO_2_. The medium was then removed, and the cells were gently rinsed three times with PBS before the addition of 200 µL pre-warmed medium containing 10% *v*/*v* of stock resazurin solution (0.15 mg/mL). The cells were then incubated for 2 h (37 °C, 5% CO_2_), and 100 µL of the medium was then transferred into a fresh 96-well plate. The fluorescence of the medium was measured at λ_ex_ 540 nm/λ_em_ 590 nm using a microplate reader (FLUOstar Omega, BMG LABTECH, Ortenberg, Germany). The measurements were normalized against the metabolic activity of control cells treated with a culture medium containing no GNUs. A negative control of cells treated with Triton X-100 (0.2% *w*/*v*, 15 min) was also included.

The possible cytotoxicity of GNUs was further evaluated with live-cell imaging using confocal microscopy. GNU suspensions at two different concentrations (0.1 and 0.2% *w*/*v*) were prepared as described above. The cells were seeded in 35 mm glass-bottomed cell culture dishes (No. 1.5 glass coverslip, IBIDI, Gräfelfing, Germany) at 3.5 × 10^6^ cells/dish and incubated for 4 days (37 °C, 5% CO_2_). Subsequently, the medium was replaced with GNU suspensions, and the dishes were incubated for 24 h at 37 °C, 5% CO_2_. Cells incubated in GNU-free medium were considered as a control. The culture medium was removed, and the cells were washed three times with HBSS prior to testing with the Live/Dead double staining kit. The assay kit was composed of the green calcein-AM and the red propidium iodide dyes, which were prepared at 2 µM and 4 µM, respectively, in PBS. The cell cultures were incubated with 100 µL of the assay solution for 15 min at 37 °C before washing twice with HBSS and imaging by confocal microscopy (LSM 880, ZEISS, Oberkochen, Germany).

## 3. Results and Discussion

### 3.1. Characterization of GNUs

**Figure 3:**
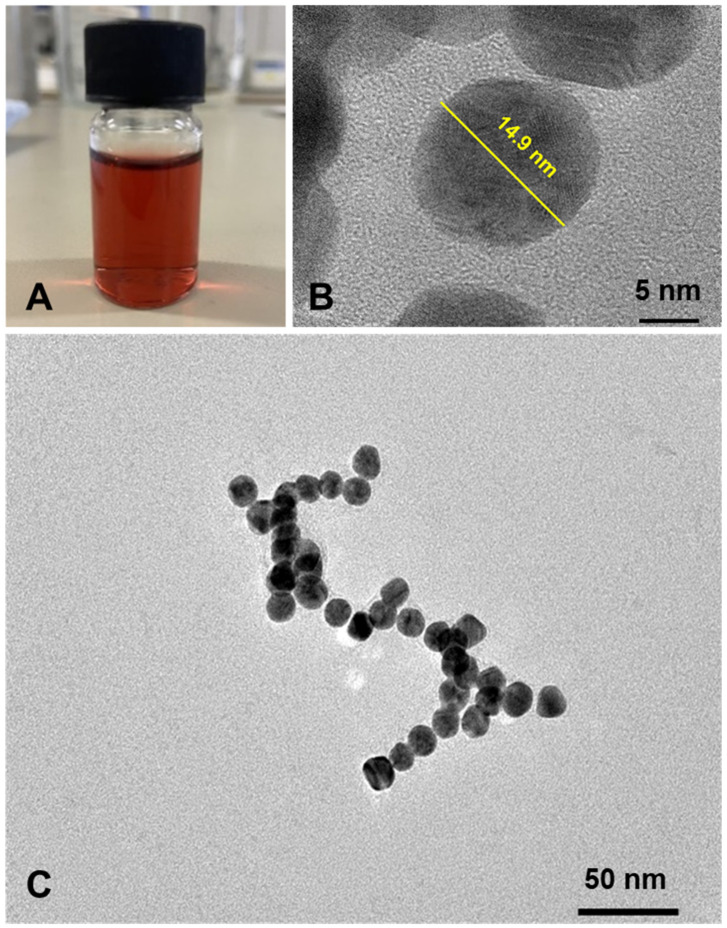
(**A**) Aqueous suspension of the citrate-capped Au seeds, (**B**) TEM image shows the spherical seeds, and (**C**) single spherical seed with higher magnification view.

A “bottom-up”, aqueous seed-mediated method based on the wet chemical reduction of a gold salt was utilized to produce monodisperse GNUs. A red/orange solution of prepared seeds was produced (Figure 3A), with the seeds being ~20.0 ± 0.5 nm in size, with a relatively narrow size distribution and negative surface charge; the zeta potential was −22.9 ± 1.0 mV according to DLS measurements. As particle size distribution is a critical property for improved regional nasal deposition, the particle diameters of the gold seeds and the subsequent GNUs were determined using more than one technique along with DLS. Imaging using TEM was consistent with the DLS results. Figure 3B,C show TEM images of monodisperse spherical particles with sizes between 15 and 25 nm. To estimate the molar concentration (*M*) of the Au seeds according to Liu et al. [29], the average number of Au^0^ atoms per particle (*N*) was first calculated in accordance with the particle diameter (*D*) as shown in Equation (1), assuming the seeds were uniformly spherical with a face-centred cubic (fcc) structure and the reaction was complete:

(1) $$N=\frac{\pi \rho D^{3}}{6m_{a}}$$

where *ρ* is the fcc gold structure density (19.3 g/mL), and *m_a_* is the atomic mass of gold (197 g/mol). For the prepared seeds, when *D* = 20 nm, *N* = 247,168 atoms/nanoparticle. The molar concentration of the colloidal gold solution was then calculated using Equation (2):

(2) $$M\;(mol\cdot L^{-1})=\frac{N_{total}}{N\times V\times N_{A}}=\frac{Au\;moles}{N\times V}$$

where *N_total_* is the total number of gold atoms in the seed solution (*N* = 1.8 × 10^19^ atoms), *V* is the reaction volume (L) and *N_A_* is Avogadro’s constant (6.02 × 10^23^ mole^−1^). Using this equation, the final molar concentration of the seed suspension would be 1.2 nM. According to Haiss et al., the size and concentration of spherical GNPs could be precisely and directly determined from UV-Vis spectra due to their unique optical properties without the need to apply calculations, except for Equation (3) [30]:

(3) M (mol·L−1)=A450ε450

where *ɛ*_450_ is the molar extinction coefficient at λ_450_, which was theoretically and experimentally verified according to [30].

Excellent agreement was obtained between the concentration calculated above, and the concentration determined from the UV-Vis spectra (*M* = 1.4 nM).

**Figure 4:**
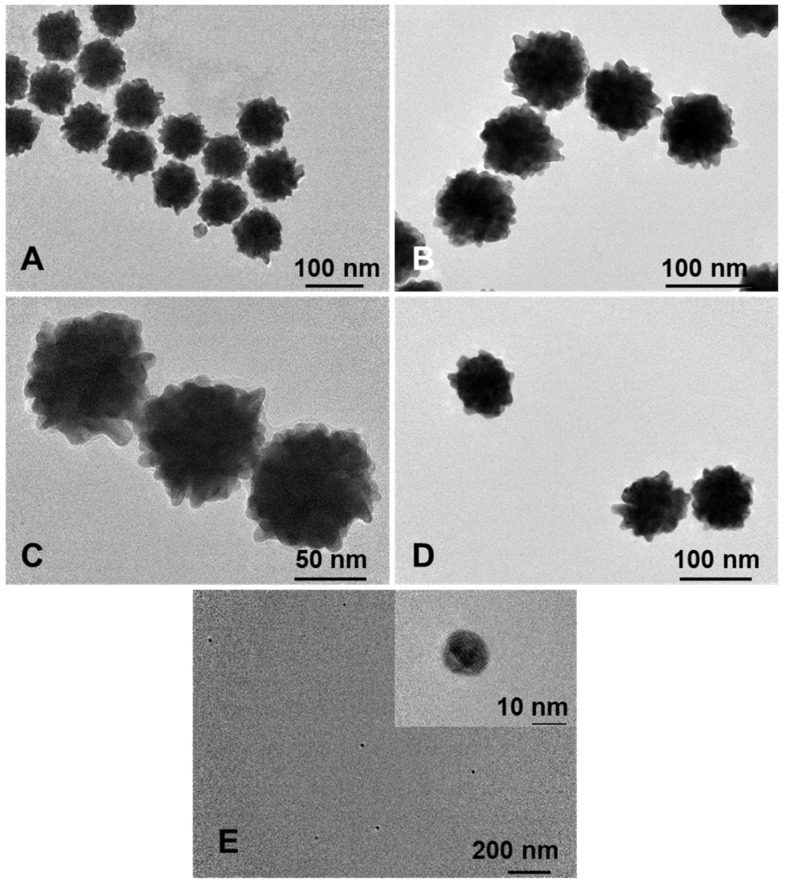
TEM images for GNUs at different DA (15 mM) volumes (**A**) 300 µL, (**B**) 600 µL, (**C**) 800 µL, (**D**) 1000 µL and (**E**) new nuclei produced at 600 µL DA with core measurement (inset).

The preparation of gold seeds was followed by the selective reduction of gold ions in the growth solution with increasing volumes of either HQ (100, 300, 500 and 1000 µL) (Figure S1) or DA (300, 600, 800 and 1000 µL). DA, a catechol, and HQ, a *p*-quinol hydroxylated aromatic, have been widely employed as reducing-cum-capping agents, which efficiently reduce Au^3+^ onto seed surfaces to generate zerovalent Au atoms and promote the formation of multibranched GNPs. The advantage of this method is that narrowly dispersed anisotropic GNPs could be successfully produced without additional conjugation steps or the need to use toxic surfactants. As expected, using incremental concentrations of HQ or DA as reducing agents during the particle growth stage resulted in blue solutions (Figure S1) and similar particle morphologies in terms of size and shape were observed (Figure S1 and Figure 4A–D). Both reductants have similar structures and mild reducing power with standard reduction potentials of E° = 0.714 V vs. NHE for HQ [23] and E° = 0.801 V vs. SHE for DA [31]. The reduction reaction of gold is complex due to the variety of species present in gold chloride solution with Au^+^ ions seeming to be the most stable [23]. Whilst reducing gold salts in the presence of metal seeds (Au^4+^/Au, E° = 1.002 V; Au^3+^/Au^+^, E° = 1.36 V; Au^3+^/Au, E° = 1.52 V) considerably differs from reducing isolated Au^+^/Au^0^ in the solution (E° = 1.83 V), the latter is more likely to be a non-spontaneous reaction with both reductants used. Likewise, the slow growth kinetics of GNPs (i.e., the rate of reduction of Au^3+^) in the presence of gold clusters results in the formation of anisotropic GNUs with a low aspect ratio [32]. While the amount of Au seeds and the HAuCl_4_ salt were kept constant, lower feeding concentrations/volumes of HQ and DA were not sufficient to generate a branched structure instead of creating a considerable number of new seeds due to the poor reaction reproducibility (stepwise Au^3+^/Au^0^ reduction), which is required to provide the growth of branches. No branched structures were produced when volumes below 300 µL of 15 mM HQ (Figure S1) or DA were added. As the volume of each reductant increased, a slight decrease in the particle aspect ratio was observed (Figure S1 and Figure 4D).

This phenomenon is similar to that observed when using a stronger reducing agent such as ascorbic acid (E° = −0.08 V), where Au^3+^ ions are rapidly reduced with no sufficient time for branch growth. These results are in agreement with those reported by Li et al., who studied the effect of different parameters on the size and shape of multibranched Au-Ag nanoparticles as a potential tool for photothermal therapy [33]. Moreover, new nuclei with approximate diameters of 10 nm were generated when volumes ≥ 600 µL of DA were added (Figure 4E), suggesting that secondary nucleation occurred, a significant obstacle when producing anisotropic metal nanostructures. Based on these data, 300 µL of 15 mM DA was chosen as the reducing agent to generate GNUs for further testing.

Du et al. suggested that when using DA as a reductant for the generation of GNPs, it not only oxidizes but also polymerizes to yield amorphous polydopamine (PDA). These PDA molecules may further interact with the surface of GNPs via gold–quinone interactions to form a PDA capping layer, which increases the stability of the particles without the requirement of an additional stabilizing agent [34]. 

The previous theory was examined in this study by preparing GNUs with and without PEG as a stabilizing agent. Noticeable aggregation occurred in some parts of the sample in the absence of PEG, confirming the contribution of stabilizing in tandem with electrostatic repulsion in maintaining a longer shelf life under the reaction conditions used in this study (Figure S2). These findings are contrary to Du et al., where PDA-coated GNPs were produced when DA was used as the reducing agent. No additional stabilizing agent was added, and the particles were stable in an aqueous solution for around 6 months [34]. The authors proposed that the DA autoxidation rate is dependent on the pH and that the PDA shell was formed when the DA reductant solution was maintained at pH 8.0, at which the dopamine–quinone species could be easily formed. This might not have been the case in the present study as varying the pH was not considered and was kept neutral at pH 6.9 (Milli-Q water). As such, DA did not act as a reducing-cum-stabilizing agent during GNU formation, and PEG was used instead to stabilize the GNUs.

**Figure 5:**
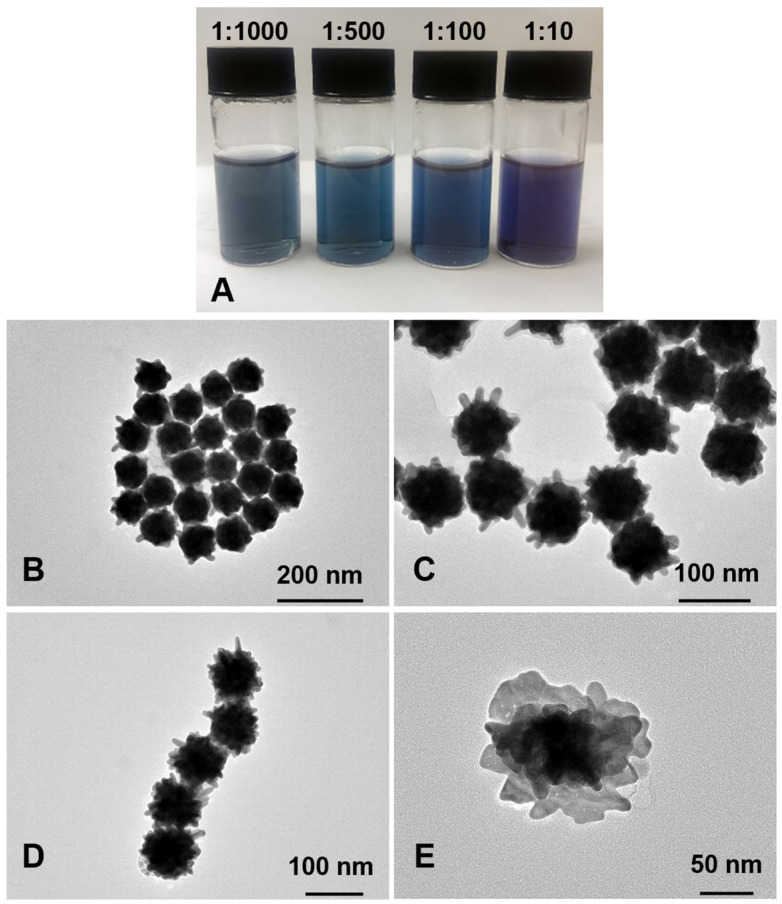
(**A**) GNU suspensions at different Ag:Au ratios and TEM images of the resulting gold nanoflowers: 1:1000 (**B**), 1:500 (**C**), 1:100 (**D**) and 1:10 (**E**).

The effect of varying Ag^+^ concentrations on GNU morphology was also examined. The inclusion of AgNO_3_ mediates the formation of anisotropic GNPs [35,36]; however, there is a debate about its precise role in the control of GNP geometry. This is mainly due to the complicated particle growth mechanism, with numerous reactants being present in the growth medium, either working in synergy or competing. Several studies have suggested that underpotential deposition is a key mechanism of the Ag^+^ shape-directing effect, where silver occupies and blocks specific facets and provokes the growth of other facets, causing anisotropic particle evolution [36,37,38]. Figure 5B–E shows TEM images of the GNUs obtained at incremental Ag:Au molar ratios and fixed concentrations of HAuCl_4_, gold seeds, DA and PEG. No significant differences were observed in terms of size, shape, and aspect ratio between samples with progressively increasing Ag^+^ concentrations (Figure 5B–D). However, an interesting purple colloid of particles with a flower-like morphology was produced at higher silver concentrations (Figure 5A; Ag:Au = 1:10). These nanoflowers were between 120 and 150 nm in diameter (Figure 5E). Zhang et al. reported nanorod-derived anisotropic morphologies when Ag^+^ was increased in the growth solution. While elongated trisoctahedral GNPs were produced in the absence of Ag^+^, dog bone-like nanorods were formed with a relatively low Ag:Au salt molar ratio (>0.02). These structures further evolved into arrow-headed nanorods when the ratio was increased. The authors proposed that the geometries generated in their study were the result of the interswitch between two main pathways, Ag underpotential deposition or Au-Ag co-deposition, where Ag and Au atoms mixed with each other at a distance far deeper from the surface atomic layer, forming Ag/Au alloy structures [38].

Similarly, Atta et al. investigated the role of increased concentrations of AgNO_3_ (30 µM–110 µM) on the growth mechanism of 6-branched gold nanostars. An increase in the spike length was observed as AgNO_3_ concentrations were increased, with a corresponding gradual red shift of the UV-Vis spectrum. A blue shift was observed when higher concentrations of AgNO_3_ (>100 µM) were employed. By tracking metallic silver deposition, it was demonstrated that Ag first acts as a stabilizing agent by saturating the deposition sites on the spikes and preventing other shape impurities. When more Ag was present, i.e., high concentrations of AgNO_3_ were used, silver was also detected at the core of the nanostars and in between spikes [37]. The authors also stated that polyhedral, not branched, NPs were formed in the absence of silver ions, which is in agreement with our attempt to produce trisoctahedral and rhombic dodecahedral particles. Both studies suggested that Ag^+^ ions at certain concentrations in the growth solution could lead to interesting, anisotropic GNU morphologies due to Ag intermixing with Au at the centre of the particles and not only as adatoms at the particle surface.

Detailed investigations using structural characterization techniques, including energy-dispersive X-ray spectroscopy, X-ray photoelectron spectroscopy and surface-enhanced Raman spectroscopy may provide clear answers to how and why these flower-like structures were produced. However, as producing monodisperse and reproducible anisotropic GNUs for aerosolization testing was the aim of this research, rather than elucidating the role of Ag^+^ in controlling GNU morphology, a 1:1000 ratio of [AgNO_3_]/[HAuCl_4_] was adopted as a GNU shape-controlling agent. 

After screening various reaction parameters to produce monomodal GNUs suitable for inhalation and, preferably, with a high aspect ratio of their branches, the optimized formulation parameters at 30 °C are shown in Table 1. The spike aspect ratio (spike length/spike base width) of the obtained particles was measured using ImageJ software, v1.53t and was found to be ≤2.0 (Figure S3). According to DLS measurements, the average hydrodynamic size for the GNUs was 98.6 ± 0.6 nm, which was further confirmed via NanoSight NS500 analysis (Figure S4), making this formulation suitable for intranasal administration with a narrow size distribution (PDI 0.06 ± 0.01), which could be observed in TEM images. This feature is desirable for a reliable interpretation of particle size-dependent nasal regional deposition and subsequent bioeffects. 

**Figure 6:**
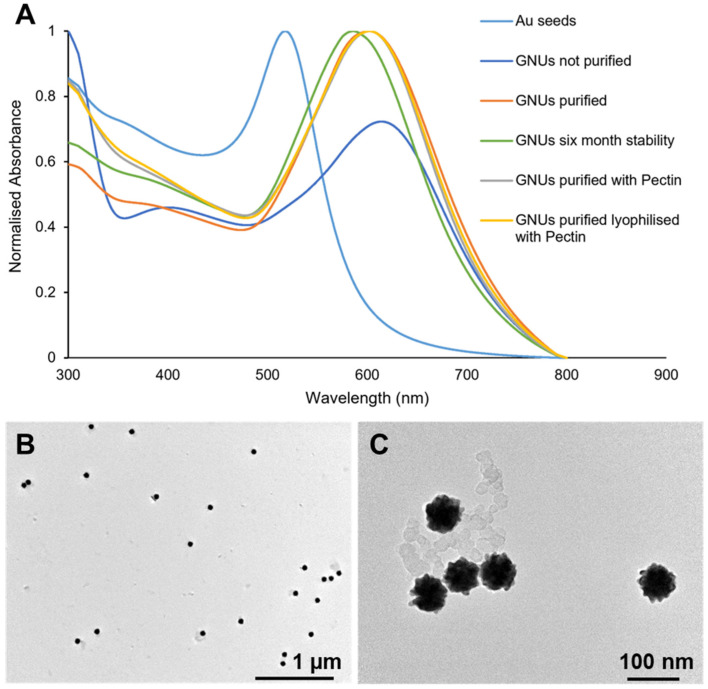
(**A**) The normalized UV-Vis absorbance spectra of the synthesized Au seeds and GNU final product showing the effect of purification, pectin addition and lyophilization procedures. (**B**,**C**) TEM images showing the stability of pectin-coated GNUs following the lyophilization process.

The UV-Vis spectra shown in Figure 6 demonstrate that the LSPR absorbance peak was red-shifted to 616 nm and 602 nm before and after purification, respectively, compared to the initial seeds at 517 nm. Increasing the aspect ratio of the GNU tips could adjust the LSPR peak towards the NIR region where biological tissues and blood are transparent, a favourable property for biomedical applications [11,14]. Although the formation of irreversible nanoparticle aggregates over time is an inherent feature of nanosuspensions which limits the functionality of the particles [39], the GNUs showed good stability over a relatively long-term storage period. An aqueous GNU suspension in sealed glass vials was stored at 4–8 °C over 6 months. As shown in Figure 6, the LSPR peak, indicative of metallic NP size and shape, only slightly shifted towards the blue region (586 nm), which could be due to occasional clustering between a few nanoparticles. The satisfactory stability profile for the GNUs can be explained by the addition of mPEG-SH chains during the synthesis. PEGylation prevents Au adatoms, i.e., Ostwald ripening, and other sorts of particle fusions in the GNP ageing process. These undesirable phenomena occur due to the large surface area and high surface energy of GNPs, with the particles tending to rearrange into more thermodynamically favourable morphologies, which result in particle aggregation and reshaping [40]. However, PEGylation did not preserve particle stability during the lyophilization process, and a collapsed lyophilizate was observed following the 16 h process. Following lyophilization, dry powder GNUs were not redispersible in water and obvious aggregates, which were resistant to breakup even after 15 min sonication, were formed. Although lyophilization is considered gentle and one of the promising approaches to convert NP suspensions into a dry powder, it may pose several stresses that result in colloidal instability via NP fusion, aggregation or content leakage for loaded particles [41]. To overcome these issues, many reports have proposed various approaches for the freezing process and formulation to yield lyophilized products, which are easy to redisperse upon reconstitution and preserve the colloidal physiochemical properties.

A simple and efficient procedure reported by Jauregui-Gomes et al. utilizing diluted pectin solution to stabilize gold nanosuspensions was adapted [24]. The addition of aqueous pectin solution to the GNU formulation resulted in a sponge-like mass following lyophilization, which was easily resuspended in Milli-Q water (to 2% *w*/*w* of the total pMDI formulation) by gentle shaking and sonication for 3 min. Figure 6 demonstrates the normalized UV-Vis spectra for the suspended GNUs upon the addition of pectin and when resuspended after lyophilization, demonstrating excellent stability. The spectra for the purified GNUs with and without pectin and following lyophilization were identical, indicating that pectin did not cause any adverse effects on the GNU morphologies, preserving them throughout the process.

Despite the homogeneous appearance of GNU–pectin dispersions, DLS data demonstrated an increase in the particle hydrodynamic diameter from 98.6 ± 0.6 nm for the as-synthesized non-lyophilized GNUs to 169.9 ± 0.8 nm for the lyophilized formulation, with a narrow size distribution (PDI < 0.22). These results were confirmed with NanoSight NS500 analysis (Figure S5), with a broad peak extending between 100 and 200 nm. Considering the data obtained with DLS and NTA techniques, the presence of some aggregates in the sample cannot be ignored. However, the larger sizes obtained could be explained by the presence of pectin (M_w_ 110–150 kDa) on GNU surfaces after reconstitution in water, which also resulted in a decrease in GNU surface charge from −10 ± 0.6 mV to −35.9 ± 0.2 mV due to the anionic polysaccharide. The stability of the pectin–GNU lyophilizate was further visualized using TEM (Figure 6). The particles exhibited morphologies similar to non-lyophilized ones. Although the pectin concentration used in this study was relatively high, testing lower concentrations or the use of other cryoprotectants was outside the scope of this work; hence, the GNU lyophilizate reconstituted in Milli-Q water with the properties shown in Table 2 was further tested for dispersion in a pMDI formulation.

### 3.2. GNU Integrity in a pMDI Formulation

In contrast to nebulizers and dry powder inhalers, pMDIs utilize a liquified gas propellant as a bulk medium that provides the energy to generate an aerosol cloud, which could enhance NP dispersion and aerosolization performance. The GNUs (0.025% *w*/*w*) were introduced into the formulation as a highly concentrated aqueous suspension along with EtOH (2% *w*/*w*) as a cosolvent and HFA134a as the propellant. No other non-volatile species were added. The use of non-volatile solvents such as H_2_O and EtOH in the developed formulation was kept to a minimum in order to reduce the proportional effect of the atomized droplet sizes due to their slow rate of evaporation and, consequently, diminished respirable deposition [42]. The effectiveness of the developed pMDI formulation was assessed for the capability to generate a gold particle aerosol with unchanged physiochemical properties and to aid the deposition of these particles in the targeted region in the nasal cavity, i.e., the olfactory region represented by Section 4T in the 3D-printed model (Figure 2).

Preliminary appearance testing of the GNU formulation was performed using transparent polyethylene terephthalate pMDI canisters. The gravitational stability and, hence, the released dose of a suspended formulation is affected by density differences between the formulation and the rest of the system. As the aqueous gold suspension was less dense than the propellant, a creaming effect occurred, and a two-phase system was generated. A homogenous suspension was obtained by manually shaking the canisters three to ten times with enough time for actuation before phase separation occurred. Spray-to-spray reproducibility was assessed as one of the U.S. Food and Drug Administration’s criteria for nasal product performance evaluation [43]. Throughout this study, the shot weights of the GNU-pMDI formulation between different canisters were consistent within the range 57–63 mg with a manual actuation force (~5.8 kg) in agreement with Doughty et al.’s observations, where in contrast to pediatric force settings, no significant differences were found in spray weights when adult-hand-spraying settings were applied [44]. 

**Figure 7:**
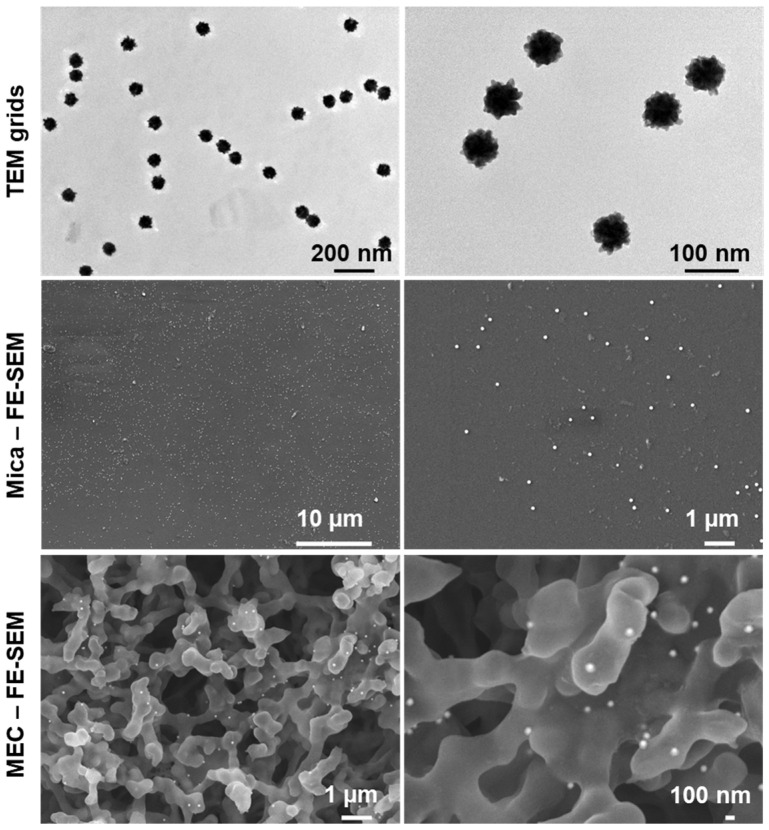
TEM and FE-SEM images showing the integrity of the atomized GNU-based pMDI. The TEM grid and the mica substrate were placed on the MEC membrane during the experiment in the glass collection apparatus.

Following atomization, the released particles were assessed for the maintenance of their morphology prior to nasal distribution testing by utilizing the apparatus shown in Figure 1B. Particles deposited on copper-coated carbon grids, mica substrates and MCE membranes were observed with TEM and FE-SEM imaging. As shown in Figure 7, not only were the size and shape of the GNUs perfectly preserved, but the particles were also highly dispersed with no clustering or aggregation observed. EDX spectra of particles collected on the receiver membrane were recorded to confirm the presence of metallic gold on the surface of the organic material (Figure S6).

### 3.3. Regional Nasal Deposition of Aerosolized GNUs

It is recognized that aerosol performance and regional deposition in the respiratory system are governed by the formulation characteristics of inhaled particulates (i.e., aerodynamic size, shape, mass, density and hygroscopicity) [42,43,44,45], the device (i.e., expansion chamber and nozzle design) [46,47,48] and patient use (i.e., administration technique and inhalation pattern) [49,50]. However, a comprehensive understanding of the role of each factor and the interactions between them are complex and yet to be elucidated. Utilizing a nasal cast enables straightforward drug deposition sampling and provides data that closely mimics aerosol performance in the human nasal cavity. As such, these in vitro tools offer the ability to optimize aerosol parameters under controlled conditions. 

HFA-based pMDIs have been widely utilized for pulmonary drug delivery, but there is little information regarding their performance for nasal drug deposition [51]. The concept of nasal pMDIs was introduced for the treatment of allergic rhinitis to overcome the limitations of aqueous pump sprays, including the loss of formulation by running down the throat or dripping from the nose and the resulting unpleasant taste and burning sensation, which limited the delivered dose and patient compliance. From a deposition perspective, pMDIs generate much smaller droplet sizes (≤5 µm) than aqueous nasal sprays, where larger particles are expected to develop inertial impaction in the nasal anterior vestibule and nasopharynx [52,53,54], whereas smaller particles (0.5–5 µm) are expected to travel further down to the lower respiratory tract [55]. The possibility of enhancing the deposition of ultra-small particles in the posterior region of the nasal cavity, where the olfactory region is located, for NTBDD utilizing a pMDI device has not yet been well investigated in vitro. This has become an attractive topic as a computational fluid dynamic model, predicting airflow behaviour and nanoscale aerosol distribution for optimal posterior nasal deposition [56,57,58].

**Figure 8:**
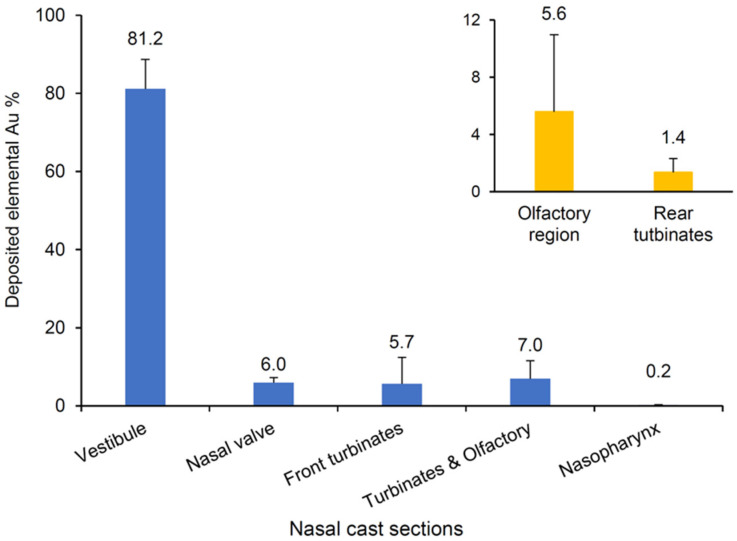
Estimated percentage of Au deposited (mean ± SD, n = 3) for GNU-pMDI in each section of the mucus-coated-nylon nasal replica determined by ICP-OES and under the following conditions: in-house actuator and a needle (1.6 × 40 mm) were used to aerosolize the particles, 30 actuations by hand with 0 L/min air flow. The inset is for the rear turbinate and olfactory region distribution.

The in vitro nasal deposition of the GNU-pMDI formulation was determined using a human nasal replica generated from an open-access source for the human nasal geometry [26] and further segmented as suggested by Hughes et al. [27] and Schroeter et al. [28] (Figure 2). A canister-to-Luer needle adaptor was used to actuate 30 shots of formulation into the cast, and the procedure was repeated in triplicate for deposition data collection. The needle (which represents the nozzle in this study) had a regular wall and bevel, which was directed into the nasal cavity to maximize deposition. As no airflow was applied, the deposition of aerosolized GNUs within the cast sections was determined by the propellant force and according to the particle aerodynamic diameters and morphology. The elemental gold recovered from each section was determined using ICP-OES, and the deposition pattern was reported as a percentage of the total Au recovery (Figure 8).

The results demonstrate that, despite the majority of the released particles (~80%) depositing in the anterior vestibule of the nasal cavity (Section 1), 13% of the GNU aerosol was able to penetrate beyond the nasal valve region and relatively even distributions were observed between the nasal valve (Section 2; 6%) and the turbinates (Section 3; 5.7% and 4; 7%) including the targeted olfactory region (Section 4T; 5.6%). 

The likely deposition profile, with the majority of the dose being restricted to the anterior nasal cavity, may be associated with agglomeration of the GNUs, resulting in larger residual particles which impacted on the anterior region of the cast and failed to diffuse through the narrow passages towards the anterior of the cavity. Perhaps more likely is the effect of the restrictive geometry of the nasal passages, which limits the full development of the aerosol plume [59] from the pMDI. The material and shape of the nozzle used (represented by the needle), along with the reduced vapour pressure of the pMDI formulation as a result of the addition of non-volatile solvents (4% *w*/*w* in total), could also have resulted in a narrow plume angle (<20°), as previously stated by Chen et al., where, for pMDI aerosols, plume geometry can be modified by the formulation vapour pressure, physicochemical characteristics of the actuator material and the nozzle geometry [60]. As such, the homogenous dispersion of the ultrafine GNUs, together with the narrow aerosol plume, allows the particles to navigate through the extremely small and convoluted nasal passages and achieve measurable dosing beyond the nasal valve. Vachhani et al. performed a numerical analysis to optimize nanoformulation delivery utilizing a particle release map, which traced particle deposition sites back to the initial injection position [57]. Under normal breathing conditions (5–20 L/min), 100 nm particles achieved 8.7% olfactory deposition within the particle size and deposition ranges of this study. Using a pMDI device, Siu et al. studied sinus targeting in the nasal cavity employing a post-functional endoscopic sinus surgery model. In comparison to a nasal spray, a pMDI produced superior overall sinus deposition (14.5% vs. 2.6%) where low-inertia particles were generated under the optimized conditions (0 L/min flow rate, 45° administration angle) [51]. 

Despite the controlled procedure utilized in the current study, in terms of the single nasal cast geometry, cast tilting, nozzle insertion depth, minor shake-fire delays and actuation force, a range of variability in the posterior delivery (4.0–13.5%) for replicate runs was observed. Such variability was also recently reported in a similar study [61]. Suspension pMDI formulations demonstrate inherent dose uniformity concerns [62]. The difference in density between suspended aqueous colloids and the continuous propellant phase may have created a heterogeneous suspension gradient over device usage. As such, in this study, a relatively high number of actuations (30 shots) were performed per run to ensure detectable concentrations in the olfactory region. Each individual run represented a different phase of the device life (i.e., beginning, middle and end) and, therefore, variability may have resulted from low- or high-dose deliveries towards the end of the canister life [49]. By increasing the gold colloid load (>0.025% *w*/*w*) within the pMDI formulation, fewer actuations per test would be required; thus, the variability between doses could be reduced. Other factors, such as the manual actuation following repeated shakes in between, and the lack of a device tip holder to ensure consistent positioning of the device tip (the beveled needle), may have also contributed to variability. The flow rate in this study was maintained at 0 L/min (i.e., breath-hold) despite the logic that nasal inspiration may aid GNU penetration deep into the nasal cavity. In fact, within the studied size range of the GNUs, inspiration is more likely to have a negative impact on their nasal deposition with more, if not most, of the particles escaping the nasal passages and depositing in the lower respiratory tract. 

The correlation between the urchin-like shape of the particles, particle density, aerosol performance and the subsequent nasal distribution could not be established in this study due to the lack of aerosol laser-based diagnostic techniques such as laser diffraction, phase Doppler anemometer, laser Doppler anemometer, and high-speed laser image analysis.

### 3.4. In Vitro Cytotoxicity of GNU Formulation

**Figure 9:**
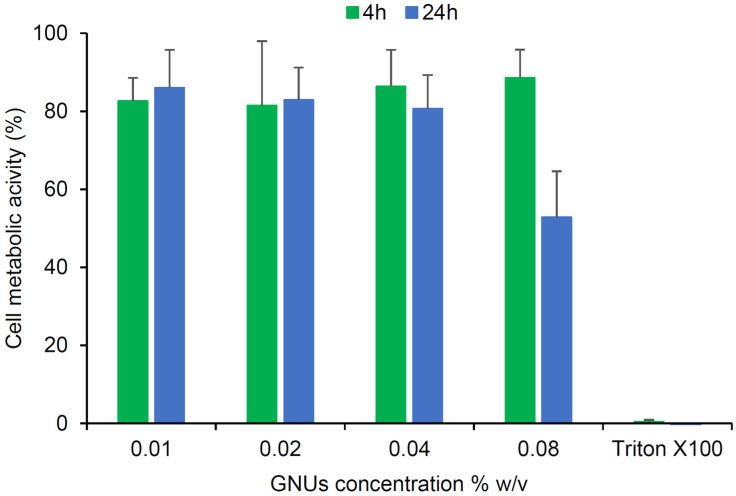
RPMI 2650 nasal cell metabolic activity (% relative to control cells) (mean ± SD, n = 6) following exposure to incremental concentrations of GNU aqueous dispersion for 4 h and 24 h.

RPMI 2650 nasal epithelial cells were used to evaluate the safety of GNUs as a potential carrier system for NTBDD after 4 and 24 h of exposure. A pre-determined mass of the pectin-based lyophilized GNUs (Table 2) was mixed with culture medium to prepare incremental concentrations of GNUs (0.01. 0.02, 0.04 and 0.08% *w*/*v*). These were then incubated with RPMI 2650 cells, and any cytotoxic effects were subsequently examined using the resazurin assay. This assay determines the metabolic activity of cells via the enzymatic reduction of resazurin to the water-soluble, cell membrane permeable, and highly fluorescent resorufin. The fluorescence intensity of the generated resorufin relative to control cells with no GNU treatment was determined. As shown in Figure 9, no harmful effects were observed for all concentrations tested, with >80% cell metabolic activity maintained after 4 h incubation. The excellent cell activity was also preserved after 24 h incubation with GNU concentrations <0.08% *w*/*v*. Similarly, catechol-functionalized gold nanoflowers (64–90 nm) were described to be biocompatible with human breast epithelial cells [11], and Wang et al. also reported high viability (>80%) of human bronchial epithelial cells that were incubated with PEGylated gold nanoparticles (spheres, ~46 nm) for 6 h, indicating their feasibility as an intranasal drug carrier according to the authors [16]. On a tissue level, it was also previously reported that rat nasal mucosae tolerated both transferosome-GNP and nanoemulsion–GNP systems, with the olfactory epithelium remaining totally intact [18].

In this study, a noticeable cytotoxic effect (~50%) occurred at the highest GNU concentration, 0.08% *w*/*v*, after 24 h incubation, which might be due to the presence of aggregated NPs formed when the colloid was introduced to the cell culture media. Such stability issues are more likely to occur when collisions between particles increase, such as in the case of high NP concentrations [63]. Although it was previously stated that GNP aggregation did not induce any toxic responses in three different cell lines (HeLa, A549 and MDA-MB-435), and the aggregate sizes reported were only 26–98 nm [64], which are smaller or equal to the particles prepared in this study. The same study reported different aggregate behaviours in terms of their uptake and subsequent accumulation in cells, with it being difficult to accurately predict due to the multitude of factors involved [64]. 

The unique shape of the GNUs may be another reason for the reduced cellular activity at elevated concentrations. Unlike gold nanorods and nanoprisms, nanospheres and nanourchins have greater interaction points with the cell surface, further facilitating the internalization and penetration process. Gallardo-Toledo et al. reported a gradual decrease in BV-2 microglial cell viability when using ≥1 nM concentrations of gold nanospheres, whereas gold nanoprisms did not cause deleterious effects at similar concentrations [14]. Comparable findings were reported by Carnovale et al., where gold particles with flat and broad interfaces were taken up in lower numbers and were better tolerated by cells than spherical ones [65]. Despite the fact that Ong et al. found that gold nanoflowers with a sphere-like shape achieved greater cellular uptake in comparison to gold nanourchins [11], the nanourchins prepared by Ong et al. were highly branched and had a higher aspect ratio compared to the GNUs presented in this study, supporting the hypothesis of facile cell internalization of the prepared particles, which may have adversely impacted the RPMI 2650 cells at high concentrations.

**Figure 10:**
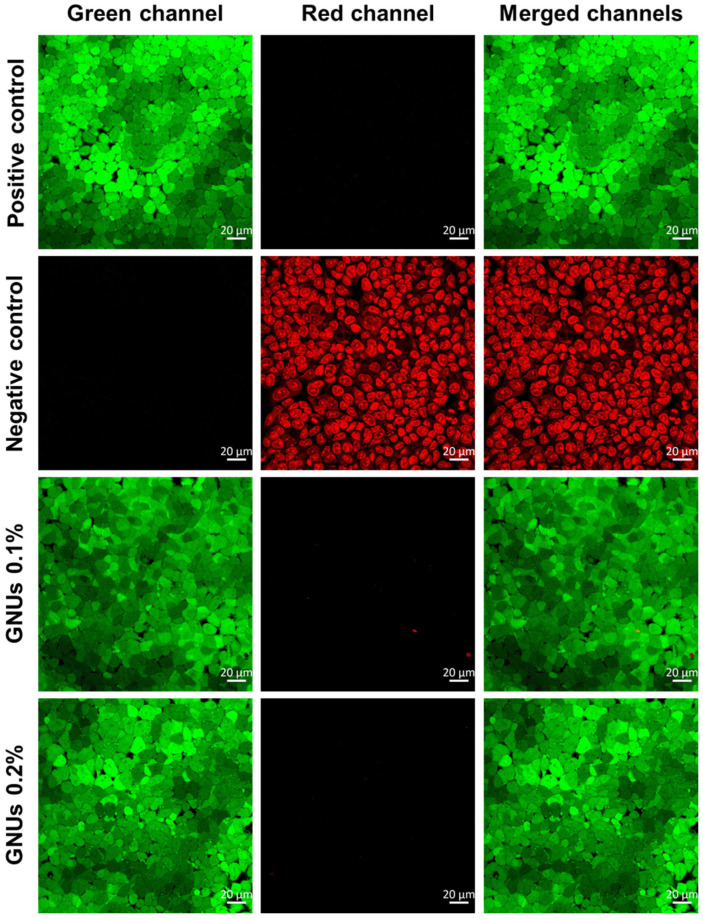
Fluorescence confocal micrographs for the viability of the RPMI 2650 nasal cells exposed to incremental concentrations of GNU aqueous dispersion for 24 h at 37 °C. Live cells are stained green, while dead cells are stained red. The positive control is untreated cells, whereas the negative control is cells incubated in 70% ethanol for 30 min.

Despite the significant yet unpredictable influence of GNU shape and size on cellular uptake and accumulation mechanisms, some variations might inhibit cellular enzymatic activity via different pathways without causing cellular death [66]. To explore this further, the nasal cells were subjected to a similar GNU treatment with two concentrations, 0.1% and 0.2% *w*/*v*, for 24 h, followed by Live/Dead staining. Interestingly, the prepared GNUs did not cause cell death, even at concentrations that are considered to be supra-physiological (2000 µg/mL), as shown in Figure 10. The RPMI 2650 nasal cell multi-layers were viable to a large extent, with very little red fluorescence caused by propidium iodide staining of nonviable cells. Instead, a uniform green fluorescence was observed, resulting from calcein-AM uptake by viable cells at both concentrations tested (0.1 and 0.2% *w*/*v*). Nonetheless, considering the contradictory reports concerning the toxicity of gold nanoparticles and the deposition of GNUs throughout the nasal cavity and not just the olfactory region, it is important for future studies to examine the possibility of unwanted off-target cytotoxicity and hypersensitivity and to take steps to ameliorate these possibilities.

## 4. Conclusions

In this study, GNUs with interesting morphology and optical properties were prepared via a facile seed-mediated chemical reaction. The effects of a number of reaction parameters on particle morphologies were investigated, namely the reducing agent, shape-producing agent and PEG functionalization. Dopamine hydrochloride or hydroquinone as reducing agents generated particles with identical urchin-like morphologies, where the aspect ratio of these GNUs was easily controlled by adjusting the added volume of both reductants. Increasing the Ag^+^ concentration as a shape-directing agent did not alter GNU morphology, but when a high Ag:Au ratio (1:10) was used, flower-like particles were formed. PEGylation was essential to preserve the particles during the synthesis, whereas a pectin matrix was required to produce a uniform lyophilized cake.

The optimized GNU formulation was incorporated as an aqueous colloid into a pressurized metered dose inhaler formulation. The GNUs were successfully released from the pMDI device and were intact and discrete following aerosolization. Furthermore, their deposition pattern was examined in a 3D-printed human nasal cast to determine the suitability of this formulation for targeting the olfactory region of the upper nasal cavity, which directly links to the brain for NTBDD applications. Up to 13% of the delivered particles were able to bypass the extremely narrow nasal valve, with deposition of up to 5.6% of the total recovered dose in the olfactory section of the cast, thus providing a proof of concept that nose-to-brain targeting may be possible using GNUs delivered using a pMDI device. Further enhancement of olfactory targeting is required to maximize the potential for NTBDD, which may be achieved via the optimization of the triple arm correlation between the formulation, device, and inhalation technique metrics. 

A further important aspect to consider for the eventual translation of this approach to intranasal or NTBDD was the potential toxicity of the GNUs. When the RPMI 2650 nasal epithelial cell model was exposed to GNUs within the concentration range used in the inhaler, no toxicity was observed in terms of metabolic activity and cell viability. This type of gold nanoparticle has proved, in other studies, to be a viable drug carrier for IN administration. Hence, the lack of toxicity observed in this study further demonstrates the potential of these nanocarriers for possible NTBDD strategies. However, the overall GNU design (size, unique shape and capping ligand) ultimately influences aerosolization performance and cellular internalization, which will need to be explored in further work.

Overall, the prepared GNUs could be utilized as a carrier core for small- as well as macro-therapeutic molecule delivery via IN administration. The tunable GNU morphologies, optical properties and excellent safety profile in nasal epithelial cells suggest the future potential for diagnostic and therapeutic tools for NTBDD. Using a pMDI as a nasal delivery device has the potential to enhance the olfactory targeting in the nasal cavity, which could result in improved drug concentrations in the brain. This could be further enhanced by particle surface functionalization with molecules specific/selective to diseased brain tissues, potentially reducing the gap to the successful treatment of CNS disorders.

## Figures and Tables

**Table 1:** The optimized formulation parameters for GNU synthesis at 30 °C.

	Reagent (Concentration)	Volume Added
Reaction medium	Milli-Q H_2_O	10 mL
Catalysts	Au seeds (1.4 × 10^−6^ mM)	60 µL
Gold salt	HAuCl_4_ (100 mM)	20 µL
Shape directing agent	AgNO_3_ (0.1 mM)	100 µL
Stabilizing agent	mPEG-SH (1.7 mM)	80 µL
Reducing agent	DA (15 mM)	300 µL
Cryoprotectant	Pectin (0.25% *w*/*v*)	0.14 mL

**Table 2:** Physicochemical characteristics (DLS measurements) for the prepared gold nanoparticles (under conditions presented in Table 1) loaded into the pMDI device.

	NP Size (nm)	PDI	Zeta Potential (mV)
Gold seeds used	20.1 ± 0.5	0.49 ± 0.003	−22.9 ± 1.0
GNUs before lyophilization	98.6 ± 0.6	0.06 ± 0.01	−10.0 ± 0.6
Reconstituted GNUs after lyophilization in water	169.9 ± 0.8	0.21 ± 0.005	−35.9 ± 0.2

## Data Availability

The original contributions presented in the study are included in the article/supplementary material, further inquiries can be directed to the corresponding author.

## Supplementary Materials

The following supporting information can be downloaded at: https://www.mdpi.com/article/10.3390/pharmaceutics16050669/s1, Figure S1: Gold nanourchin suspensions generated with different volumes of hydroquinone; Figure S2: The effect of PEGylation on GNUs; Figure S3: Measurement of selected spike aspect ratios on the optimized GNUs using ImageJ software; Figure S4: Size distribution of GNUs in comparison to the initial seeds measured with NanoSight NS500; Figure S5: Size distribution of lyophilized pectin-based GNUs in comparison to the as-synthesized, non-lyophilized GNFs measured with the NanoSight NS500; Figure S6: FE-SEM-EDX analysis for element composition of gold nanourchins.
