# Supplementary material for: Gold Nanoparticles: Tunable Characteristics and Potential for Nasal Drug Delivery
Source: Pharmaceutics. 2024 May 16;16(5):669. doi: 10.3390/pharmaceutics16050669 (PMC11125093; doi:10.3390/pharmaceutics16050669)
Supplement: Supplementary file 1 [file pharmaceutics-16-00669-s001.zip › pharmaceutics-2912077-supplementary.pdf]

Supporting Information for:

Gold Nanoparticles: Tunable Characteristics and Potential for Nasal Drug Delivery

Aida Maaz[1], Ian S. Blagbrough[1] and Paul A. De Bank[1,2,3*]

[1] Department of Life Sciences, University of Bath, Bath BA2 7AY, UK
[2] Centre for Therapeutic Innovation, University of Bath, Bath BA2 7AY, UK
[3] Centre for Bioengineering & Biomedical Technologies, University of Bath, Bath BA2 7AY, UK
***** Correspondence: p.debank@bath.ac.uk; Tel.: +44(0)1225384017

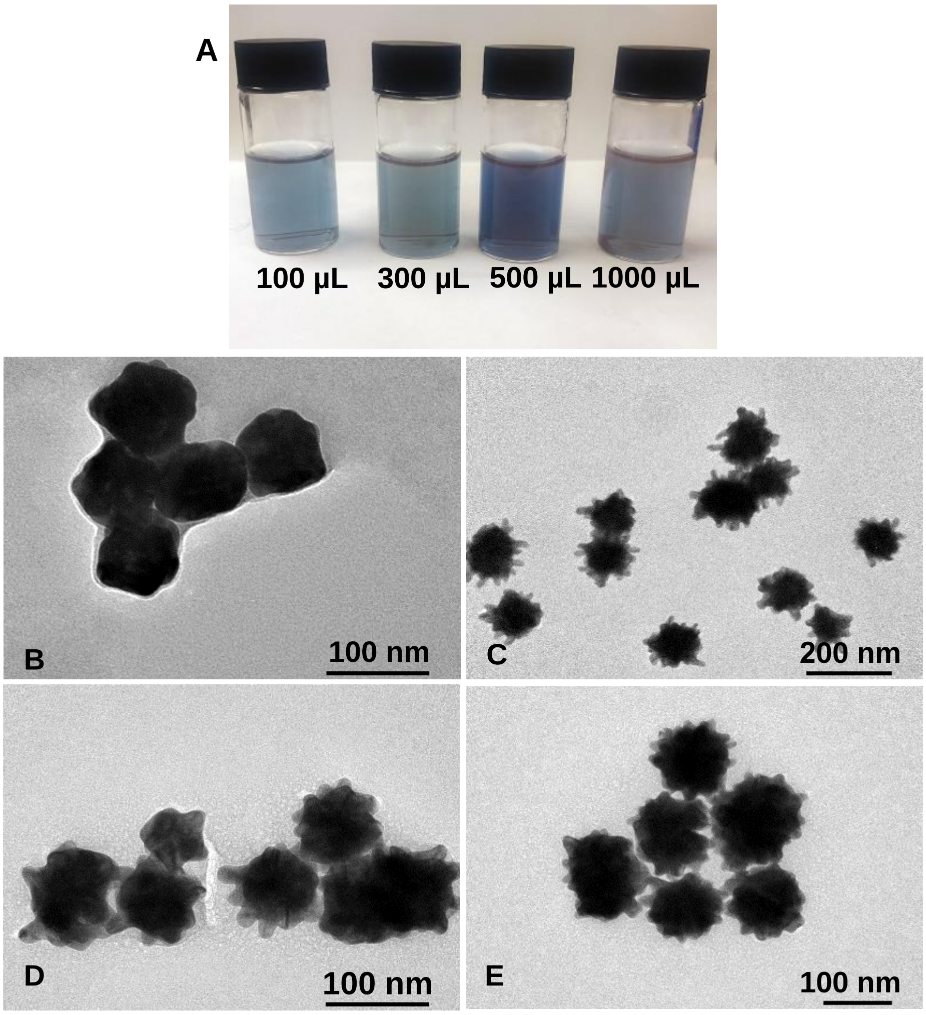

Figure S1. (**A**) Gold nanourchin suspensions generated with different volumes of 15 mM hydroquinone and (**B-E**) TEM images of the resulting GNUs using 100 µL (**B**), 300 µL (**C**), 500 µL (**D**) and 1000 µL (**E**) of HQ.

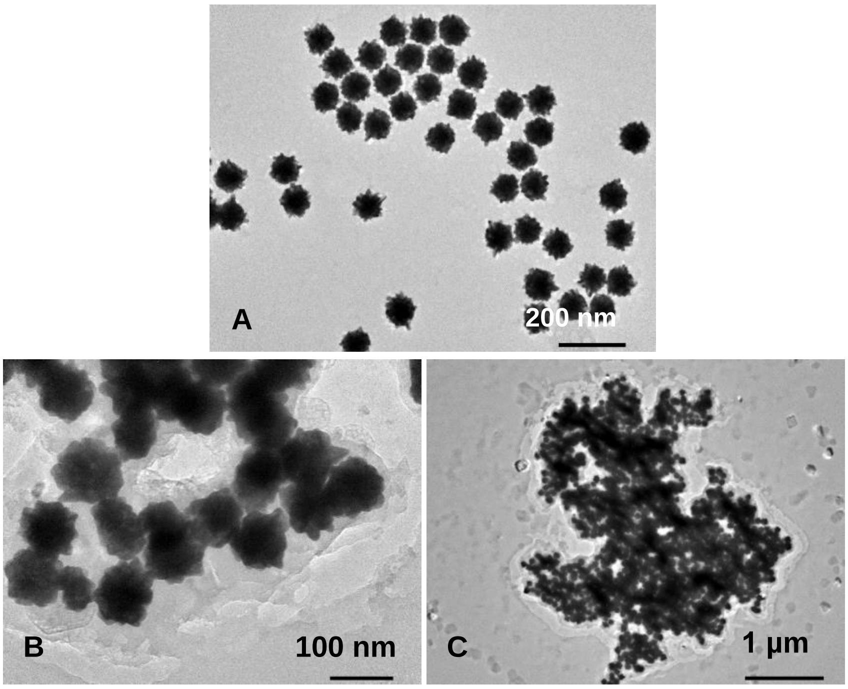

Figure S2. The effect of PEGylation on GNUs. TEM images of PEGylated (A) and non-PEGylated GNUs (B and C).

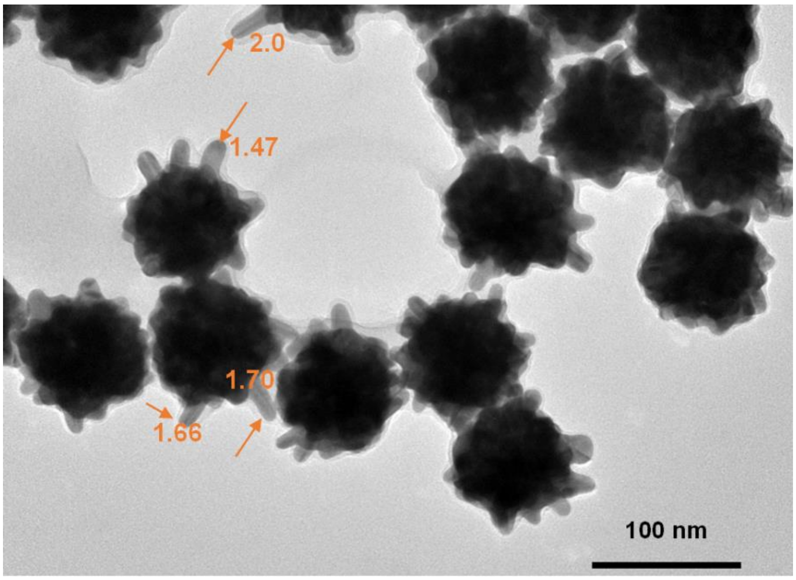

Figure S3. Measurement of selected spike aspect ratios on the optimized GNUs using ImageJ software.

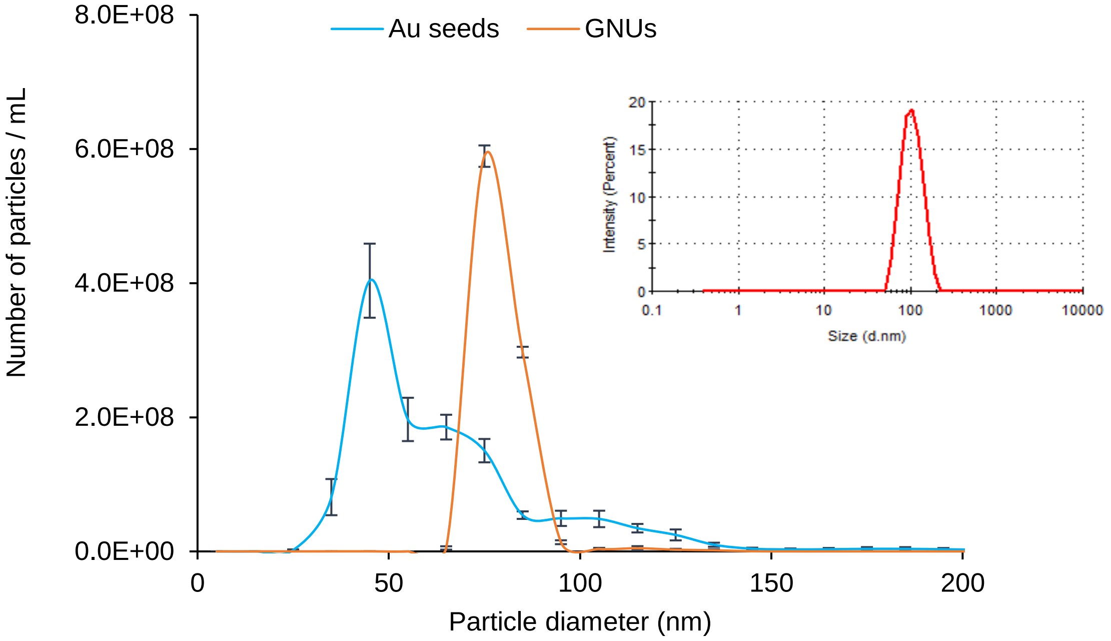

Figure S4. Size distribution of GNUs in comparison to the initial seeds measured with a NanoSight NS500. Inset: Histogram of GNU particle size measured by DLS.

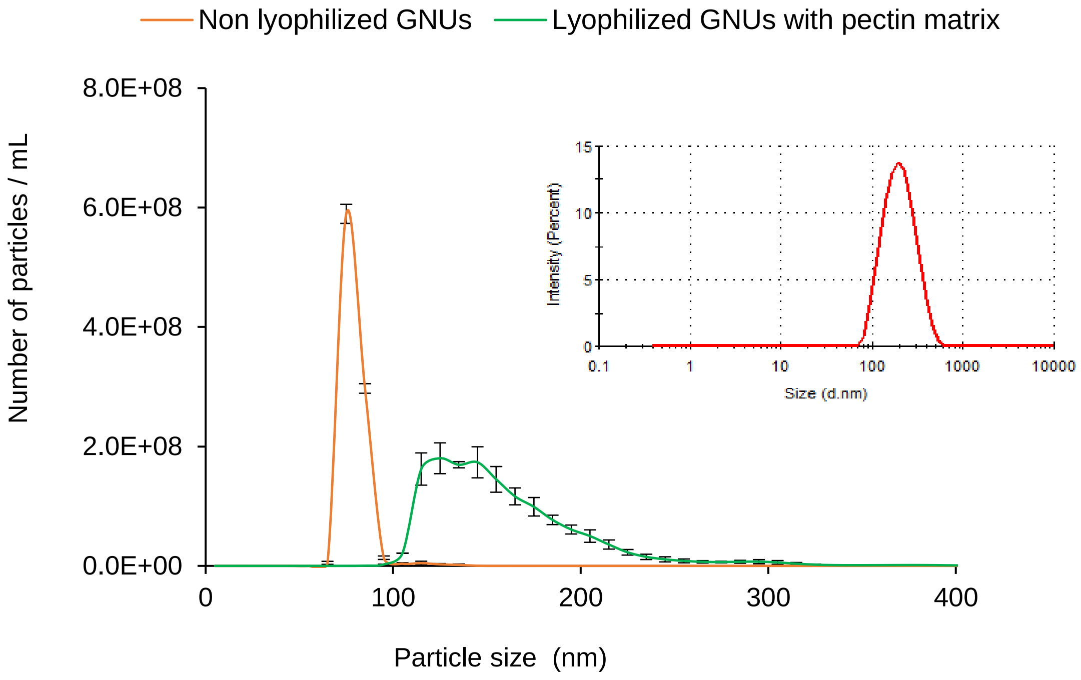

Figure S5. Size distribution of lyophilised pectin-based GNUs in comparison to the as-synthesised, non-lyophilised GNFs measured with the NanoSight NS500. Inset: Histogram of GNU particle size post-lyophilization measured by DLS.

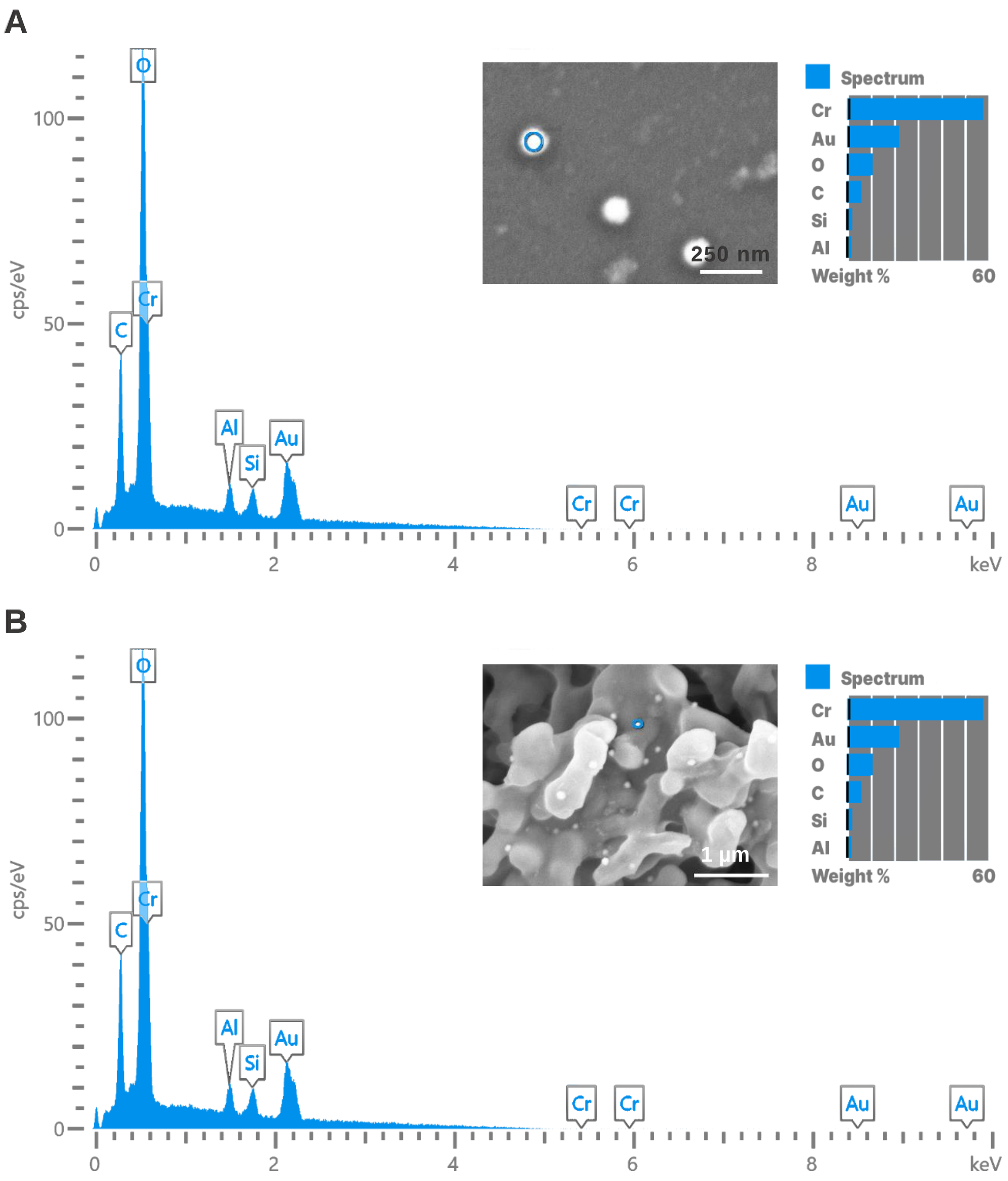

Figure S6. FE-SEM-EDX analysis for element composition of gold nanourchins. A) GNUs deposited on mica, and B) GNUs on a mixed cellulose ester membrane.
